# Exploring mortality patterns in Pakistani children and adolescents aged 5–19: evidence from a national verbal and social autopsy study

**DOI:** 10.7189/jogh.16.04195

**Published:** 2026-09-11

**Authors:** Sajid Bashir Soofi, Imran A Chauhadry, Sidrah Nausheen, Enas Mariam, Muhammad Umer, Shanila Nooruddin, Muhammad Atif Habib, Arjumand Rizvi, Henry D Kalter, Robert E Black, Zulfiqar A Bhutta

**Affiliations:** 1 Centre of Excellence in Women and Child Health, Aga Khan University, Karachi, Pakistan; 2 Department of Paediatrics & Child Health, Aga Khan University, Karachi, Pakistan; 3 Department of Obstetrics & Gynaecology, Aga Khan University, Karachi, Pakistan; 4 Institute for Global Health & Development, Aga Khan University, Karachi, Pakistan; 5 Institute for International Programs, Bloomberg School of Public Health, Johns Hopkins University, Baltimore, Maryland, USA; 6 Centre for Global Child Health, The Hospital for Sick Children, Toronto, Ontario, Canada

**Keywords:** adolescent mortality, verbal autopsy, Pakistan, cause of death, child mortality, verbal and social autopsy

## Abstract

**Background:**

While under-five mortality has declined by approximately 50% globally since 2000, mortality among older children and adolescents has fallen by only 31% since 1990. The age group 5–19-year-olds constitute over a third of Pakistan’s population, yet estimates of mortality in this population, which are necessary for identifying preventable causes of death and developing interventions to improve health outcomes, have been limited. We aimed to investigate cause-specific mortality in children aged ≥5 years using verbal and social autopsy (VASA) to identify patterns and health risks across these age groups.

**Methods:**

We used the multistage stratified cluster sampling frame of the Pakistan National Nutrition Survey 2018, which covered 115,600 households from urban and rural areas. Through retrospective mortality surveillance, we identified 1,322 deaths among 5–19-year-olds in these households in the preceding three years. We then obtained data on their deaths from the household members through VASA interviews, after which a trained paediatrician and two computerised methods assigned specific causes of death to each case.

**Results:**

We completed VASA interviews for 1,322 deaths, out of which 29.8% were among children aged 5–9 years, 24.4% among young adolescents (10–14 years), and 45.8% among older adolescents (15–19 years). Overall mortality was higher among males (59%) than females (41%). Infectious diseases, non-communicable diseases (NCDs), and accidents/injuries were the three main causes of death, with the contribution of infectious diseases declining, and that of NCDs and injuries increasing with age. Maternal causes, particularly obstetric complications, contributed significantly to mortality among female adolescents.

**Conclusions:**

We found age- and gender-based differences in mortality causes among the 5–19-year population in Pakistan. Targeted efforts are needed to sustain the gains achieved among the under-five population by extending mortality reduction strategies that focus on age-specific risks for older children and adolescents.

The United Nations’ ‘Leave No One Behind’ agenda, integral to the 2030 Sustainable Development Goals, emphasises promoting health and well-being for all age groups [1]. Though most child mortality occurs among those under five years of age, deaths among older children and adolescents remain substantial and require greater public health focus. In 2022, an estimated 0.9 million adolescents (10–19 years) died primarily from preventable or treatable causes [2]. While global mortality rates are the lowest among older children (5–9 years), they rise again in young (10–14 years) and older adolescents (15–19 years) [3]. While under-five mortality has declined globally by nearly 50% since 2000, it has dropped by only 31% among older children and adolescents since 1990 [2]. Significant regional disparities in mortality rates persist, with the highest rates of mortality in this age group reported in sub-Saharan Africa, followed by Oceania (excluding Australia and New Zealand) and Central and Southern Asia [2].

The 5–9-year mortality rate, denoting deaths per 1,000 children aged 5–9 years among those who have survived to age five, is higher in Pakistan (4.1 per 1,000 children) than the global average (3.4 per 1,000 children) [2]. While 5–9-year-olds experience higher mortality compared to children under the age of five years, they face unique risks besides infectious diseases and congenital anomalies, such as injury-related deaths (*e.g.* road traffic accidents (RTAs) and drowning) [3,4]. The 10–14-year mortality rate is the probability of dying between ages 10 and 15, expressed per 1,000 ten-year-olds [5]. In this age group, mortality causes have shifted in recent decades, with infectious diseases now accounting for 49% of deaths globally (vs 30% from injuries and 20% from non-communicable diseases) [2]. Mortality among 10–14-year-olds is now dominated by infectious diseases (49%), followed by injuries (30%) and non-communicable diseases (NCDs) (20%) [2]. Lastly, the mortality rate for adolescents aged 15–19 reflects the deaths 15-year-olds before age 20 [5]. Here, males face a 1.6-fold higher risk of death (5.4 per 1,000 children) compared to females (3.5 per 1,000 children) [2]. Gender differences are evident in terms of causes of death: NCDs (39%) lead among females, followed by injuries (37%), infectious diseases (17%), and maternal causes (4%), whereby injuries dominate among males (48%), followed by NCDs (24%) and infectious diseases (22%) [2].

These figures indicate a need for targeted interventions to address the causes and determinants of mortality in this age group. According to the 2023 census, 37% of Pakistan’s population falls within the 5–19 age group [6]. Social determinants such as poverty, poor sanitation, disaster-prone environments, food insecurity, inadequate infrastructure, political instability, and gender inequality significantly contribute to mortality among school-age children and adolescents in the country [4,7]. Evaluating mortality in this demographic is, therefore, essential for identifying preventable causes and implementing effective interventions that could improve health outcomes.

However, data gaps on causes of death (CoDs) limit efforts in addressing these issues. Specifically, Pakistan lacks a comprehensive, high-quality national vital registration system for CoD data that covers the 5–19-year age group, and further faces issues related to incomplete death registration, poor medical certification, and limited verbal autopsies (VAs) [8]. Existing data from the World Health Organization (WHO) portals and the Global Burden of Disease analyses offer partial or modelled information [9]. The Pakistan Demographic Survey reports mortality rate data disaggregated by age groups and sex, but fails to provide detailed CoD information [10]. Hospital records, meanwhile, are limited in coverage and often encompass NCDs or injuries only [8], while local research focuses on infant and under-five child mortality, without age stratification beyond five years [4].

We aimed to address these gaps by using a nationally representative sample and the Verbal and Social Autopsy (VASA) tool to estimate CoDs among individuals aged 5–19 years, categorised into five-year age groups to highlight age-specific mortality patterns and risks [10]. The VASA, which combines structured caregiver interviews on symptoms and circumstances of death, is particularly valuable in the absence of robust registration systems [11]. In Pakistan, VA has been validated for its diagnostic accuracy in identifying the causes of maternal deaths, stillbirths, and neonatal deaths and is considered a reliable method for determining the CoD [12–15].

## METHODS

We conducted a cross-sectional household survey with retrospective ascertainment using the Pakistan National Nutrition Survey (PNNS) sampling frame provided by the Pakistan Bureau of Statistics [6]. Following this, we conducted VASA interviews in households where deaths had occurred among children aged 5–19 years within the three years before the survey. Full methodological details, including sampling, questionnaire adaptation, training, and analytical procedures, are reported in the accompanying study protocol [16].

### Sampling

The PNNS was conducted from April 2018 to February 2019 across four provinces of Pakistan, the Islamabad Capital Territory, and the two administrative areas of Azad Jammu Kashmir and Gilgit Baltistan, thereby encompassing both urban and rural areas. The sampling frame from PBS was based on the 2017 population census, comprised of 168,943 enumeration blocks or primary sampling units, each comprising 200–250 households. A total of 5,780 primary sampling units (20 households each), comprising 115,600 households, were selected across 156 districts for PNNS. The district-level representative sample size was calculated using the prevalence of nutrition-related indicators [17].

### Household selection for VASA interviews

Using the NNS 2018 sampling frame of 5,780 primary sampling units, our team conducted a mortality census in 4,475 clusters covering 502,933 households (~3.4 million individuals) to identify deaths. The survey teams administered a mortality questionnaire to identify deaths among individuals aged 5–19 years that occurred in the three years preceding the survey. Deaths among 5–19-year-olds were ascertained through the same household enumeration used across the wider VASA study; a separate target sample size was not calculated specifically for this age group. A total of 1,318 VASA interviews were completed, corresponding to approximately 3.3% precision for the most common cause of death closely matching the 3% design target used for stillbirths, neonatal, and child deaths.

### Training of study staff

The VASA team, comprising a male supervisor and female interviewers, underwent a comprehensive seven-day classroom training programme followed by two days of field training, focusing on interviewing techniques and questionnaire concepts. Senior faculty members of the Aga Khan University (AKU) facilitated five workshops at the provincial level, while a VASA expert from JHU held a simulated workshop training for the CoD analysis teams on using the ICD-10 to ascertain CoDs [18]

### Data collection tool – VASA Questionnaire

We used the VASA questionnaire to determine CoDs. This questionnaire integrates the WHO’s VA tool with the Johns Hopkins Bloomberg School of Public Health – Institute for International Programs (JHU/IIP) Social Autopsy (SA) tool, thus allowing a deeper insight into both medical and social factors contributing to mortality [19]. The verbal component involves a structured interview with family members or caregivers to gather information about symptoms, signs, and circumstances surrounding the death [20], while the social component collects information about social, cultural, religious, and healthcare system factors [19]. We adapted the questionnaire after pilot testing to ensure cultural sensitivity and alignment with local norms, translating it into the local language (*i.e.* Urdu) and back into English to verify content validity. We focused exclusively on the VA component of the VASA tool for CoD identification. The SA component was beyond the scope of this analysis; therefore, the findings presented here are limited to cause-specific mortality and do not capture the social, behavioural, and health-system factors contributing to mortality.

### Data collection

We revisited households that were identified with deaths among individuals aged 5–19 years during the mortality survey for VASA interviews. We approached the caregivers in these households, informed them about the study's purpose, and invited them to participate after they provided informed consent. The AKU managed the data control system and supervised the VASA database to ensure data quality.

### Data analysis

We descriptively summarised the results, disaggregated by sex (male and female) and WHO age category (older children: 5–9 years; young adolescents: 10–14 years; and older adolescents: 15–19 years). We reviewed responses to both the narrative section of the VA questionnaire and related sections containing closed-ended questions about illness signs and symptoms to determine CoDs. A review team comprising paediatricians and physicians trained in CoD analysis conducted the analysis, reviewing the data from each interview and classifying the biological CoDs. Two physicians independently reviewed each case; in cases of disagreement, the case was referred to a specialist physician for final review. Additionally, we employed the InterVA-5 and InSilicoVA computer-coded verbal autopsy (CCVA) analysis to determine CoDs [21,22], despite their lower reliability compared to physician-certified verbal autopsy (PCVA), due to its public health surveillance and scalability for programmatic use.

We classified CoDs using a hierarchical framework aligned with the WHO’s VA standards and the Global Burden of Disease cause list, adapted for adolescents (15–19 years) [23,24]. We grouped the causes into four broad categories: infectious diseases, NCD, accidents and injuries, and maternal causes. We also constructed an ‘undetermined causes’ category for cases where data were insufficient for reliable cause attribution.

Descriptive statistics were used to summarise the data. Categorical variables were presented as frequencies and percentages, whereas continuous variables were reported as means and standard deviations (SDs). Statistical analyses were performed using STATA version 18 (StataCorp LLC, College Station, Texas, USA).

## RESULTS

We identified 1,322 deaths of children and adolescents through the mortality survey and conducted VASA interviews to investigate the underlying CoDs. Among these, 394 (29.8%) pertained to child deaths (5–9 years), 322 (24.4%) were young adolescents (10–14 years), and 606 (45.8%) were older adolescents (15–19 years). Overall mortality was higher in males than females, at almost 59% compared to 41% (**Table 1**). Most deaths occurred at home (39.8%) and hospitals (31.3%), while 11.7% occurred during travel to healthcare facilities.

**Table 1:** Deaths among children and adolescents aged 5–19 years by sex and place, n (%)

	Total	5–9 years	10–14 years	15–19 years
**Sex**				
Male	779 (58.9)	229 (58.1)	200 (62.1)	350 (57.8)
Female	543 (41.1)	165 (41.9)	122 (37.9)	253 (42.0)
**Place of death**				
Home	525 (39.7)	176 (44.7)	145 (45.0)	204 (33.7)
Hospital	412 (31.2)	113 (28.7)	99 (30.7)	200 (33.0)
Other places	208 (15.7)	53 (13.5)	39 (12.1)	116 (19.1)
On route to a health provider or facility	154 (11.6)	47 (11.9)	36 (11.2)	71 (11.7)
Don't know	16 (1.2)	1 (0.3)	3 (0.9)	12 (2.0)
Other health providers or facilities	7 (0.5)	4 (1.0)	0 (0.0)	3 (0.5)

The mother was the primary respondent in 87.4% of VASA interviews. The average recall period was approximately one year: 1.08 (SD = 1.16) for child deaths, 1.2 (SD = 1.61) for young adolescents, and 1.06 (SD = 1.16) for old adolescents.

### Household characteristics

The average household density, defined as the number of individuals per room, was nearly 4 for all households (**Table 2**). Less than half (45.6%) of the households were within 30 minutes of the nearest health facility. Almost all (91.0%) households had access to improved water sources, and nearly 68.5% had access to improved toilet facilities.

**Table 2:** Household and maternal characteristics of children and adolescents aged 5–19 years*

	5–9 years	10–14 years	15–19 years
**Household characteristics**			
Household density (people/rooms), x̄ (SD)	4.3 (2.3)	4.1 (2.2)	3.5 (2.0)
Improved sources of drinking water	350 (88.8)	292 (90.7)	565 (93.2)
Improved toilet facilities	248 (62.9)	220 (68.3)	450 (74.3)
**Time taken to reach the nearest health facility, in hours**			
0–0.5	173 (43.9)	141 (43.8)	296 (48.8)
0.5–1	119 (30.2)	91 (28.3)	168 (27.7)
>1	80 (20.3)	70 (21.7)	110 (18.2)
Do not know	22 (5.6)	20 (6.2)	32 (5.3)
**Average time taken to reach nearest health facility, x̄ (SD)**	1.3 (3.1)	1.6 (5.4)	1.2 (3.0)

SD – standard deviation, x̄ – mean

*Variables are presented as n (%) unless specified otherwise.

### Causes of mortality

Through the InterVA5 and InSilicoVA analysis, we identified similarities and variations in the CoDs across the five-year mortality groups. The typical leading CoDs among individuals aged 5–19 years included accidents and injuries, infectious diseases, diarrhoeal disorders, and NCDs. Complications related to maternal health outcomes were another CoD among females aged 15–19 years. There was an age-related transition in CoDs, with infectious diseases dominating in younger children, but giving way to NCDs, accidents and injuries among older groups. In late adolescence, RTAs predominated among males and maternal complications among females.

### Children (5–9 years old)

Of the 394 deaths reported in children aged 5–9 years, 229 (58.0%) were males and 165 (42.0%) were females. According to the InterVA5 and InSilico analyses, the leading CoDs among children aged 5–9 years were infectious diseases (40.2%), followed by accidents and injuries (29.2%) and NCDs (11.8%) (**Table 3**). Diarrhoeal disorders (both infectious and non-infectious) accounted for 7.1% CoDs among 5–9-year-olds.

**Table 3:** CoDs in children aged 5–9 years by VA methods

	InterVA5, n (%)	InSilicoVA, n (%)	CCVA, %*	PCVA, n (%; 95% CI)
**All infectious diseases**	151 (38.32)	166 (42.14)	40.23	119 (30.02)
HIV	35 (8.88)	15 (3.81)	6.35	
Pneumonia	24 (6.09)	25 (6.35)	6.22	26 (6.6; 4.5–9.5)
Tuberculosis	10 (2.54)	7 (1.78)	2.16	6 (1.52; 0.7–3.4)
Measles	6 (1.52)	9 (2.28)	1.90	23 (5.84; 3.9–8.6)
Malaria	3 (0.76)		0.38	
Other (including dengue, acute abdomen, and typhoid)	73 (18.53)	110 (27.92)	23.22	64 (16.24; 12.9–20.2)
**Accidents and injuries (including self-harm)**	107 (27.15)	123 (31.22)	29.19	121 (30.7; 26.3–35.5)
**NCDs**				
All	41 (10.42)	52 (13.2)	11.80	85 (21.57)
Cancers and neoplasms	5 (1.27)	5 (1.27)	1.27	12 (3.04; 1.7–5.3)
Congenital malformations		1 (0.25)	0.13	
Other	36 (9.15)	46 (11.68)	10.41	73 (18.53; 15.0–22.7)
**Diarrheal disorders**	30 (7.61)	26 (6.6)	7.11	25 (6.35; 4.3–9.2)
**Severe malnutrition**	8 (2.03)	11 (2.79)	2.41	9 (2.28; 1.2–4.3)
**Undetermined**	38 (9.64)	15 (3.8)	6.73	8 (2.03; 1.0–4.0)
**Other and unspecified external CoD**	19 (4.82)	1 (0.25)	2.54	27 (6.85; 4.7–9.8)

CCVA – computer-coded verbal autopsy, CI – confidence interval, CoD – cause of death, HIV – human immunodeficiency virus, NCD – non-communicable disease, PCVA – physician-certified verbal autopsy, VA – verbal autopsy

*Average of InterVA5 and InSilicoVA.

The PCVA analysis identified infectious diseases (30.0%) and accidents and injuries (30.7%) as the primary CoDs in this age group, followed by NCDs (21.6%). Conversely, diarrhoeal diseases (6.4%) were the least common CoD. The predominant infectious diseases recognised as CoDs were pneumonia, measles, and diarrhoea. Accidents and injuries were primarily attributed to RTAs, drowning, electrocution, and falls. The NCDs in this age group included neoplasms, heart disease, and kidney disease. A small proportion of deaths remained unclassified, with 2% categorised as undetermined CoD and 7% attributed to other or unspecified causes of death.

### Young adolescents (10–14 years old)

The distribution of CoDs among individuals aged 10–14 differed by gender (Table S1 in the **Online Supplementary Document**). According the InterVA5 and InSilicoVA analyses, the leading CoDs among females were infectious diseases (41.0%), followed by accidents and injuries (23.8%), NCDs (16.4%), and maternal CoDs (9.0%). However, the PCVA identified NCDs (33.6%) as the primary CoD in females in this age group, followed by infectious diseases (26.3%) and accidents and injuries (23.0%). As for PCVA, maternal causes contributed to 4.9% CoDs among females of this age group.

The primary causes identified by CCVA in males were accidents and injuries (42.5%), followed by infectious diseases (33.3%) and other NCDs (11.8%). The PCVA also identified accidents and injuries (41.5%) as the primary CoD among males in this age group, followed by NCDs (29.5%) and infectious diseases (15.0%).

The major NCDs identified as CoDs in this age group were cancer, heart disease, and liver disease. The leading CoDs among accidents and injuries were RTAs, drowning, and falls, while electrocution, firearm injuries, and violence were less common. The primary infectious diseases included meningitis/encephalitis, acute abdomen, and other unspecified infections.

### Older adolescents (15–19 years old)

We further found differences in CoDs between males and females among 15–19-year-olds (Table S2 in the **Online Supplementary Document**). Analysis using InterVA5 and InSilicoVA identified maternal causes as the leading CoD among older adolescent females (38.5%), followed by NCDs (22.5%), accidents and injuries (17.7%), and infectious diseases (16.4%). Obstetric haemorrhage was the most common CoD among maternal causes (19.4%), followed by pregnancy-related sepsis (11.3%) and pregnancy-induced hypertension (5.3%).

In contrast, the PCVA findings indicated NCDs as the predominant CoD among females (30.4%), followed by maternal causes (26.9%) and accidents and injuries (16.4%). The major CoDs among maternal causes were found as pregnancy-induced hypertension (10.5%), obstetric haemorrhage (9.4%), and pregnancy-related sepsis (2.3%). A total of 2.7% of the deaths were attributed to pre-existing medical conditions which were aggravated by pregnancy. For males, the leading CoDs identified by CCVA were accidents and injuries (57.4%), followed by NCDs (21.4%) and infectious diseases (15.1%). A comparable trend was observed in the PCVA findings, where accidents and injuries remained the leading CoD among males (52.3%). However, deaths from NCDs (26.0%) were slightly higher, while those from infectious diseases were lower (9.1%).

The most common injuries leading to death in this age group were RTAs, followed by falls and self-harm. Cancer was the most frequent CoD among NCDs, followed by cardiovascular diseases and kidney disease. Tuberculosis emerged as the primary CoD among infectious diseases.

## DISCUSSION

We investigated causes of mortality among 5–19-year-olds in Pakistan, who constitute 37% of the country’s population [6]. The gender analysis revealed higher mortality among males (59.0%) than females (41.0%), aligning again with global patterns [10]. Overall, CoDs clustered into three categories: infectious diseases, NCDs, and accidents/injuries.

Infectious diseases were the leading CoDs among 5–9-year-olds, which is in line with trends observed among under-five children, but at lower levels. The main CoDs were diarrhoeal infections, pneumonia, and measles, aligning with global data [9]. The risk of injuries also increased with increasing mobility in this age group; key contributors included drowning, electrocution, RTAs, and falls. The NCDs, particularly neoplasms, also began to appear as contributors to CoDs.

We observed a lower contribution of infectious diseases and a higher contribution of NCDs and injuries to CoD among 10–14-year-olds, consistent with the epidemiological transition from communicable to non-communicable causes of death observed globally [9]. We also noted significant gender-specific differences, whereby NCDs remained the leading CoD among females (33.6%), followed by infectious diseases (26.3%) and accidents and injuries (23.0%). These findings align with global evidence indicating malaria, neoplasms, and lower respiratory tract infections as major causes of mortality in young adolescents [9]. However, accidents and injuries dominated in males (41.5%), consistent with global estimates that identify road injuries as the leading CoD in adolescents [9]. These gender differences highlight the need to tailor interventions to each gender.

This gender gap was more pronounced in older adolescents (15–19 years). Specifically, NCDs were predominant among females (30.4%), followed by maternal causes (26.9%), accidents and injuries (16.4%), and infectious diseases (13.3%). This pattern aligns with global data, which lists NCDs as the leading CoD in this demographic, but contrasts with South Asian estimates, where self-harm is the primary cause among adolescent girls [9]. Our results highlight the persistent vulnerability of Pakistani adolescents to early pregnancy complications. Obstetric haemorrhage emerged as the leading maternal CoD, consistent with the Pakistan Maternal Mortality Survey 2019 [25]. This implies the need for adequate reproductive health services and access for this age group. In contrast, accidents and injuries accounted for most deaths in males (52.3%), followed by NCDs (26.0%) and infectious diseases (9.1%). This pattern follows global estimates, where road traffic injuries, violence, and self-harm predominate as adolescent male CoDs [9]. The high injury burden, largely stemming from RTAs, electrocution, and falls, reflects risk-taking behaviours and poor safety measures, and thereby remains largely preventable.

We found clear differences in mortality patterns across age bands. Infectious diseases, accidents and injuries, and NCDs were the primary CoDs among 5–9-year-olds, with infectious diseases accounting for the largest share; their relative contribution was lower in older age bands, with infectious diseases accounting for 30.02% of CoDs among 5–9-year-olds, 19.3% among 10–14-year-olds, and 10.9% among 15–19-year-olds, a pattern consistent with global age-related differences in infectious disease burden [3,9]. This shift reflects infection control efforts targeting children under five in low-income countries, with slower progress in reducing infections among older age groups [26]. The major contributors in the 5–19 age group were diarrhoeal infections and pneumonia, which again aligns with global estimates [27,28]. Pneumonia was the most common specific infectious CoD overall, followed closely by tuberculosis and measles, which differs from global estimates where malaria typically ranks third [27]. Moreover, NCDs accounted for a growing share of mortality with age, also consistent with global estimates [29]. Neoplasms were the leading NCD cause, particularly among females aged 10–14 years [9].

Accidents and injuries accounted for a significant portion of CoDs, particularly among adolescents, who face greater risks from street exposure, vehicle use, and interpersonal violence [30]. We identified RTAs as the leading CoD among male adolescents, a finding consistent with both global and regional trends [3,9]. Previous research showed that adolescent RTAs accounted for 12% of all RTA deaths in Pakistan [31]. This rate is driven by factors that exacerbate road hazards, creating unsafe conditions and increasing crashes and fatalities [32]. Beyond RTAs, adolescents were frequently involved in sports-related, firearm, and occupational injuries, while younger children more often suffered accidental falls, drowning, or electrocution, which have lower fatality rates, explaining the heavier mortality burden from injuries in adolescents compared to younger children.

Pregnancy and childbirth complications were significant CoDs among females aged 15–19 years, which is consistent with both global and regional data [33]. Obstetric CoDs were a major contributor in our analysis; haemorrhage, hypertensive disorder, and sepsis, in turn, account for more than half of maternal deaths at the global level [34]. Despite a decline in adolescent fertility from 56 births per 1,000 in 2000 to 44 births per 1,000 in 2019, rates remain high in many low- and middle-income countries, where over 30% of girls marry before the age of 18. Early marriage often leads to early childbearing, placing mothers aged 10–19 at higher risk of eclampsia and infections than women aged 20–24, resulting in higher fatality rates.

Deaths among 5–19-year-olds for which caregivers had incomplete symptom recall, or where rare conditions could not be identified, were labelled as ‘unspecified’. In contrast, ‘undetermined’ deaths had enough information, but the symptoms did not match any defined cause, leaving them unexplained despite detailed investigation.

### Strengths and limitations

A key strength of our study is that the data collection spanned across Pakistan, ensuring a generalisable and representative sample. We recruited trained staff from local communities, ensuring adherence to cultural norms and the use of regional languages. Several limitations, however, should be noted. First, we did not validate the PCVA against the gold standard of diagnostic autopsy. There were variations in results between the CCVA and PCVA methods, although the latter is considered more reliable. Second, there is a possibility of recall bias due to the three-year reference period; however, due to the nature of the recalled event (*i.e.* the death of a child), this is less likely to have occurred. Additionally, since determining CoD relies on reported symptoms, there might be an overlap across different conditions, leading to a possible misclassification of CoD. Lastly, our ability to provide structured psychological support to bereaved respondents was limited; this may have reduced disclosure of sensitive information (*e.g.* self-harm or interpersonal violence) and could lead to underestimation of these causes.

## CONCLUSIONS

We analysed CoDs among 5–19-year-olds with age- and gender-based disaggregation. The leading causes were infectious diseases, injuries and accidents, and NCDs. A notable shift emerged with age, as the mortality burden transitioned from communicable diseases to injuries and NCDs. These insights can inform future planning and enhance service delivery, particularly in resource-constrained settings. We also note a need for a multisectoral approach that prioritises education, strengthens health systems, implements road safety regulations, and enhances law enforcement, as well as for reproductive and other health interventions targeting adolescents specifically, to effectively address the challenges faced by our study populations.

## Additional material


Online Supplementary Document
