# Supplementary material for: Exploring mortality patterns in Pakistani children and adolescents aged 5–19: evidence from a national verbal and social autopsy study
Source: J Glob Health. 2026 Sep 11;16:04195. doi: 10.7189/jogh.16.04195 (PMC13564922; doi:10.7189/jogh.16.04195)
Supplement: Supplementary file 1 [file jogh-16-04195-s001.pdf]

Supplement to: Soofi SB, Ahmed I, Nausheen S, Mariam E, Umer M, Nooruddin S, Habib MA, Rizvi A, Kalter HD, Black RE, Bhutta ZA. Exploring mortality patterns in Pakistani children and adolescents aged 5–19: evidence from a national verbal and social autopsy study. J Glob Health. 2026;16:04195.

Table S1. Causes of death in children aged 10–14 years in males and females by various verbal autopsy methods

	**Females**					**Males**				
	**InterVA**	**InSilicoVA**	**CCVA***	**PCVA**		**InterVA**	**InSilicoVA**	**CCVA***	**PCVA**	
	**Frequency (%)**	**Frequency (%)**		**Frequency (%)**	**95% CI**	**Frequency (%)**	**Frequency (%)**		**Frequency (%)**	**95% CI**
**All Infectious diseases**	**47 (38.6)**	**53 (43.4)**	**40.98%**	**32(26.30)**		**63 (31.5)**	**70 (35)**	**33.25%**	**30 (15.0)**	
HIV	14 (11.5)	5 (4.1)	7.79%	-	-	15 (7.5)	6 (3.0)	5.25%	-	
Pneumonia	3 (2.5)	2 (1.6)	2.05%	3 (2.5)	0.8-7.4	5 (2.5)	9 (4.5)	3.50%	2 (1.0)	0.2-3.9
TB	3 (2.5)	4 (3.3)	2.87%	3 (2.5)	0.8-7.4	10 (5.0)	7 (3.5)	4.25%	4 (2.0)	0.7-5.2
Measles				1 (0.8)	0.1-5.7	3 (1.5)	3 (1.5)	1.50%	2 (1.0)	0.2-3.9
Malaria				1 (0.8)	0.1-5.7	2 (1.0)	1 (0.5)	0.75%	1 (0.5)	0.1-3.5
Infectious diseases	27 (22.1)	42 (34.4)	28.28%	24 (19.7)	13.5-27.8	28 (14)	44 (22.0)	18.00%	21 (10.5)	6.9-15.6
**Accidents and injuries**	**29 (23.8)**	**29 (23.8)**	**23.77%**	**28 (23)**	**16.3-31.3**	**80 (40)**	**90 (45.0)**	**42.50%**	**83 (41.5)**	**34.8-48.5**
**All NCDs**	**21 (17.3)**	**19 (15.6)**	**16.39%**	**41 (33.6)**		**24 (12)**	**23 (11.5)**	**11.75%**	**59 (29.5)**	
Cancers & neoplasms	3 (2.5)	3 (2.5)	2.46%	12 (9.8)	5.6-16.6	4 (2.0)	2 (1.0)	1.50%	16 (8.0)	4.9-12.7
Diabetes mellitus	3 (2.5)	1 (0.8)	1.64%	1 (0.8)	01-5.7	5 (2.5)	1 (0.5)	1.50%	4 (2.0)	0.7-5.2
Other NCDs	15 (12.3)	15 (12.3)	12.30%	28 (23)	16.3-31.3	15 (7.5)	20 (10.0)	8.75%	39 (19.5)	14.6-25.6
**Maternal CoD**	**10 (8.2)**	**12 (9.9)**	**9.02%**	**1 (0.8)**	**0.1-5.7**					
	Breakdown of all causes related to maternal mortality									
Abortion-related death	3 (2.5)		1.23%							
Ectopic pregnancy		1(0.8)	0.41%							
Obstetric haemorrhage	2 (1.6)	3 (2.5)	2.05%	1 (0.8)						
Obstructed labour	1 (0.8)		0.41%							
Other and unspecified maternal CoD	1 (0.8)		0.41%							
Pregnancy-related sepsis	3 (2.5)	8 (6.6)	4.51%							
Diarrheal disorders	4 (3.3)	4 (3.3)	3.28%	7 (5.7)		7 (3.5)	8 (4.0)	3.75%	7 (3.5)	1.7-7.2
Severe malnutrition	2 (1.6)	2 (1.6)	1.64%	2 (1.6)		3 (1.5)	5 (2.5)	2.00%	3 (1.5)	
Other and unspecified external CoD	(4.1)	3 (2.5)	3.28%	6 (4.9)		11 (5.5)	1 (0.5)	3.00%	15 (7.5)	
Undetermined†	4 (3.3)		1.64%	5 (4.1)		12 (6.0)	3 (1.5)	3.75%	3 (1.5)	

CCVA – Computer-coded verbal autopsy, PCVA – Physician-certified verbal autopsy

*Average of InterVA5 and InSilicoVA
†When three physicians did not agree on the cause of death, it was classified as undetermined. Computer-coded VA labeled a cause of death as 'undetermined' when the verbal autopsy data is incomplete, conflicting, or lacks enough patterns to assign a probable cause.

Table S2. Causes of death in children aged 15–19 years in males and females by various verbal autopsy methods

	**Female**					**Male**				
	**InterVA5**	**InSilicoVA**	**CCVA***	**PCVA**		**InterVA5**	**InSilicoVA**	**CCVA***	**PCVA**	
	**Frequency (%)**	**Frequency (%)**		**Frequency (%)**	**95% CI**	**Frequency (%)**	**Frequency (%)**		**Frequency (%)**	**95% CI**
**Maternal CoD**	**95 (37.1)**	**102 (39.9)**	**38.48 %**	**69 (26.9)**	21.9-32.7					
	break down of all causes related to maternal mortality									
Abortion-related death	6 (2.3)	1 (0.4)	1.37%	2 (0.8)						
Anaemia of pregnancy		2 (0.8)	0.39%							
Ectopic pregnancy		2 (0.8)	0.39%							
Obstetric haemorrhage	57 (22.3)	41 (16.0)	19.14 %	24 (9.4)						
Obstructed labour		1 (0.4)	0.20%	1 (0.4)						
Other and unspecified maternal CoD	2 (0.8)		0.39%							
Other specific maternal causes			0.00%	2 (0.8)						
Pre-existing medical conditions exacerbated by pregnancy			0.00%	7 (2.7)						
Pregnancy-induced hypertension	17 (6.6)	10 (3.9)	5.27%	27 (10.5)						
Pregnancy-related sepsis	13 (5.1)	45 (17.6)	11.33 %	6 (2.3)						
**Accidents and injuries (including self-harm)**	**45 (17.6)**	**46 (18.0)**	**17.77 %**	**42 (16.4)**	**12.3-21.5**	**197 (56.3)**	**205 (58.6)**	**57.43 %**	**183 (52.3)**	**47.0-57.5**
**All NCDs**	**64 (25)**	**51 (20)**	**22.46 %**	**78 (30.4)**		**74 (21.1)**	**76 (21.8)**	**21.43 %**	**91 (26)**	
Cancers & neoplasms	27 (10.5)	16 (6.3)	8.40%	18 (7.0)	4.5-10.9	28 (8.0)	22 (6.3)	7.14%	30 (8.6)	6.1-12.0
Other NCDs	37 (14.5)	35 (13.7)	14.06 %	60 (23.4)	18.6-29.0	46 (13.1)	54 (15.5)	14.29 %	61 (17.4)	13.8-21.8
All Infectious diseases	37 (14.5)	55 (21.5)	17.97 %	34 (13.3)		48 (13.8)	58 (16.5)	15.14 %	32 (9.1)	-
HIV	10 (3.9)	5 (2.0)	2.93%	1 (0.4)	0.1-2.7	17 (4.9)	5 (1.4)	3.14%	-	-
TB	7 (2.7)	5 (2.0)	2.34%	8 (3.1)	1.6-6.1	6 (1.7)		0.86%	5 (1.4)	0.6-3.4
Pneumonia	5 (2.0)	7 (2.7)	2.34%	3 (1.2)	0.4-3.6	9 (2.6)	15 (4.3)	3.43%	6 (1.7)	0.8-3.8

Malaria	3 (1.2)	1 (0.4)	0.78%	-	-	-	-	-	1 (0.3)	0.0-2.0
Measles	-	-	-	-	-	-	-	-	-	-
Other Infectious diseases	12 (4.7)	37 (14.4)	9.57%	22 (8.6)	5.7-12.7	16 (4.6)	38 (10.8)	7.71%	20 (5.7)	3.7-8.7
Diarrheal disorders	4 (1.6)		0.78%	1 (0.4)	0.1-2.7	5 (1.4)	6 (1.7)	1.57%	4 (1.1)	0.4-3.0
Severe malnutrition	1 (0.4)	1 (0.4)	0.39%	5 (2.0)	0.8-4.6	1 (0.3)	1 (0.3)	0.29%	-	-
Other and unspecified external CoD	2 (0.8)	1 (0.4)	0.59%	6 (2.3)	1.1-5.1	14 (4.0)	1 (0.3)	2.14%	36 (10.3)	-
Undetermined†	8 (3.1)		1.56%	12 (4.7)	2.7-8.1	11 (3.1)	3 (0.9)	2.00%	4 (1.1)	-

CCVA – Computer-coded verbal autopsy, PCVA – Physician-certified verbal autopsy

*Average of InterVA5 and InSilicoVA

†When three physicians did not agree on the cause of death, it was classified as undetermined. Computer-coded VA labeled a cause of death as ‘undetermined’ when the verbal autopsy data is incomplete, conflicting, or lacks enough patterns to assign a probable cause.
